# Comparative Pharmacokinetics of Sustained-Release versus Immediate-Release Melatonin Capsules in Fasting Healthy Adults: A Randomized, Open-Label, Cross-Over Study

**DOI:** 10.3390/pharmaceutics16101248

**Published:** 2024-09-25

**Authors:** Shefali Thanawala, R. Abiraamasundari, Rajat Shah

**Affiliations:** 1 Nutriventia Limited, Mumbai 400069, Maharashtra, India; rajat@nutriventia.com; 2 SpinoS Life Science Research and Private Limited, Thudiyalur, Coimbatore 641029, Tamil Nadu, India; abirami@spinoslifescience.com

**Keywords:** melatonin, pharmacokinetics, nutraceutical, sleep disorders, sustained release

## Abstract

**Background:** Exogenous melatonin, a nutraceutical for maintaining a healthy sleep–wake cycle and managing sleep disorders, requires large, repeated doses due to its low bioavailability and short half-life. This necessitates the development of a sustained-release formulation with a longer half-life and sustained plasma concentration. Therefore, exogenous novel 5 mg sustained-release melatonin capsules (Melatonin-SR, test product) were formulated. **Methods:** This open-label cross-over study compared the pharmacokinetics (maximum concentration [C_max_], time to reach C_max_ [T_max_], area under the curve [AUC], and elimination half-life [t_1/2_]) and the safety of Melatonin-SR with 5 mg immediate-release melatonin capsules (Melatonin-IR, reference product) after single-dose oral administration in healthy fasting adults. **Results:** Sixteen participants (aged 18–45 years) were randomized (1:1) to receive either Melatonin-SR or Melatonin-IR in two periods with a 7-day washout period. Melatonin-SR reported a lower C_max_ (11,446.87 pg/mL) compared to Melatonin-IR (22,786.30 pg/mL). The mean T_max_ of Melatonin-SR and Melatonin-IR was 1.26 h and 0.87 h, respectively. The mean t_1/2_ of Melatonin-SR (5.10 h) was prolonged by five-fold compared to Melatonin-IR (1.01 h). One adverse event (vomiting) was reported following the administration of the Melatonin-IR. **Conclusions:** Melatonin-SR resulted in higher and sustained plasma melatonin concentrations for an extended period and was well-tolerated. Hence, Melatonin-SR may be a promising nutraceutical for maintaining healthy sleep.

## 1. Introduction

Sound sleep is crucial for maintaining health in humans. Sleep disturbances (acute) or sleep disorders (chronic) may predispose the individual to the development and progression of major illnesses and neural changes such as depression and low mood [1]. Sleep disruptions impact the continuity, quality, and quantity of sleep and can lead to stress responsivity, somatic pain, a reduced quality of life, emotional distress, and mood disorders, as well as cognitive, memory, and performance deficits; over a prolonged period of time, these can contribute to medical issues such as hypertension, dyslipidemia, cardiovascular disease, weight-related issues, metabolic syndrome, type 2 diabetes mellitus, and colorectal cancer [2,3,4,5,6].

Insomnia, a chronic sleep disorder, is characterized by persistent difficulty encountered in either the induction or maintenance of sleep for at least one month [7]. Recent evidence from the United States of America indicates that approximately 13% of older adults had frequent insomnia and 18.1% had poor-quality sleep [8]. Moreover, according to the National Center for Health Statistics report for 2020, overall, 14.5% of adults had trouble falling asleep and 17.8% of adults had trouble staying asleep [9]. In the United Kingdom, around 34% of children (aged 7–16 years) and 64% of young people (aged 17–23 years) had a sleep problem three or more times over the previous seven nights [10]. The prevalence of insomnia is also estimated by various studies conducted on Asian populations. A cross-sectional study from India reported that 33% of individuals have insomnia [11].

The role of melatonin (N-acetyl-5-methoxytryptamine, a chronobiotic hormone produced by the pineal gland) in sleep regulation and maintaining the sleep–wake cycle is well established. It primarily influences the body's internal circadian rhythm through interaction with the suprachiasmatic nucleus, which is the central circadian pacemaker, to align bodily functions with the light/dark cycle of the surrounding environment [12]. Being considered a crucial endogenous synchronizer of the human biological clock, external melatonin is widely used in the management of insomnia and other sleep disorders, jet lag, and shift work normalization.

Several studies have reported varied pharmacokinetic profiles of melatonin. Following oral administration, melatonin is rapidly metabolized through first-pass hepatic metabolic processes such as hydroxylation and conjugation and is eliminated from the body through urine [13]. After oral administration, its time to reach C_max_ (T_max_) has been reported to be approximately 50 min, while the elimination half-life (t_½_) was about 45 min. The bioavailability of melatonin ranges from 9% to 33% following oral administration [14]. Melatonin shows variations in its overall pharmacokinetic behavior in terms of absorption, metabolism, and elimination. Many factors such as age, caffeine consumption, smoking habits, oral contraceptive use, feeding patterns in lactating mothers, and the coadministration of drugs have been reported to have an impact on melatonin pharmacokinetics [15,16,17].

Despite being an effective ingredient for restoring a healthy sleeping cycle, melatonin’s short elimination half-life combined with a high degree of variability in pharmacokinetic parameters highlights the need for modified release formulations that offer the continuous release of active compounds over a longer time period. Thus, novel sustained-release formulations of melatonin are required that can release a certain amount of the active compounds (up to 50%) immediately, thereby mimicking the endogenous melatonin profile, followed by a continuous and gradual release for a prolonged time to avoid the peak and trough effect, which normally occur with exogenous products having an immediate-release profile. Such sustained-release formulations are expected to provide elevated plasma melatonin levels for a longer duration of time, thus allowing it to exert its action for a longer duration and thus facilitating healthy sleep. Besides being a sleep promoter, melatonin exhibits several other intriguing effects (such as antioxidant, anti-inflammatory, and antiapoptotic effects) that may be potentiated by the sustained-release formulation [18].

Melotime^TM^, a nutraceutical supplement, was formulated as 5 mg sustained-release capsules of melatonin (Melatonin-SR, test product) to provide a uniform release and maintenance of melatonin levels in the plasma during a sleep period of 8 h with a gradual tapering down effect without causing a spillover of sleep during the waking hours. The present study aimed to compare the pharmacokinetic profile and the safety and tolerability of Melatonin-SR capsules with immediate-release 5 mg melatonin (Melatonin-IR, reference product) capsules after single-dose oral administration in healthy adults under fasting conditions.

## 2. Materials and Methods

### 2.1. Study Design

This was an open-label, balanced, randomized, single-dose, two-treatment, two-sequence, two-period, two-way cross-over, oral comparative pharmacokinetic study conducted in healthy adults under fasting conditions at Spinos Life Science and Research Private Limited, Tamil Nadu, India, from 28 August 2022 to 7 September 2022.

### 2.2. Study Population Eligibility

Participants aged between 18 and 45 years with a body mass index (BMI) ranging between 18.50 to 29.99 kg/m^2^, with no evidence of underlying disease during screening as documented by laboratory screening values (which were within normal limits as per the physician’s or principal investigator’s discretion), considered healthy based on the medical history, physical examination (including but not be limited to an evaluation of the cardiovascular, gastrointestinal, respiratory, musculoskeletal and central nervous systems, and gynecological examination and breast examination for women), vital sign assessments, 12-lead electrocardiogram (ECG) and chest X-ray, who were nonsmokers or ex-smokers (defined as one who completely stopped smoking for at least in the past three months of study initiation), and willing to comply with all the requirements of the study protocol and those instructed by the study personnel were included in this study. Participants having an allergy to melatonin, a history of any pre-existing disease (which might compromise the hemopoietic, gastrointestinal, renal, hepatic, cardiovascular, musculoskeletal, respiratory, central nervous system, or any other body system), presence of diabetes mellitus, psychiatric disorders, renal or hepatic impairment, history of alcohol addiction or abuse, regular consumption of caffeine and xanthine products, or having used tobacco containing products for at least a day prior to the study were excluded. Additional exclusion criteria included the use of any prescription medications/any hormonal agent 14 days prior to the study, use of any over the counter medicinal products 7 days prior to the study, use of hormone replacement therapy 6 months prior to the study, donation of blood within 90 days prior to the study, unusual diet, a positive result of a urine test for drug abuse, a positive test for alcohol, pregnant, or breastfeeding women, or a history of difficulty in swallowing. Grapefruit and its juice and poppy-containing foods were restricted 48 h prior to and during the study period.

### 2.3. Study Procedure

After obtaining informed consent from the participants, the screening evaluations were performed to determine the eligibility of participants within 21 days before check-in period 1. The participants were housed at the clinical facility for at least 36.00 h pre-dose to 24.00 h post-dose for period 1 and period 2. Both the periods were separated by a 7-day washout period. The total study duration was 11 days.

The test products were Melatonin-SR capsules containing 5 mg melatonin (Nutriventia Limited, Mumbai, Maharashtra, India), and the reference products were Melatonin-IR capsules containing 5 mg melatonin (Huanggang Saikang Pharmaceutical Co., Ltd., Huanggang City, China). Both products were administered to the participants following a randomization schedule (using block randomization method with block size of 4) created using R software, version 4.0.4 (Auckland, New Zealand) (by an independent statistician). During each period, in the morning between 8:00 a.m. to 8:14 a.m., the participants received Melatonin-SR or Melatonin-IR after overnight fasting of at least 10 h. As per the randomization schedule, the single oral dose of Melatonin-SR or Melatonin-IR was administered along with 240 mL of drinking water to the participants in an upright sitting position (under the supervision of the trained study personnel) and participants were instructed to remain in the same position for four hours post-dosing. Participants were not allowed to lie down during the restriction period. Thereafter, participants were allowed to move freely during the remaining part of the study. However, they were not allowed to be engaged in any kind of strenuous activity during the study period. Drinking water was allowed to be consumed ad libitum during the study period except it was restricted 1 h pre-dose and 1 h post-dose. Standardized meals were provided at −34.00, −24.00, −20.00, −16.00, and −12.00 h of each period pre-dose and at 4.00, 8.00, and 12.00 h after dosing. Standardized lighting conditions were maintained in the clinical phase unit to suppress the production of endogenous melatonin. This standardization included housing the participants in a windowless room on the dosing day and was maintained throughout the entire blood sampling period in both the treatment periods with all light readings between 500 to 600 Lux. Light levels were measured four times in a day in both the study periods (7:00 to 8:00 a.m., 12:00 noon to 1:00 p.m., 5:00 to 6:00 p.m., and 11:00 p.m. to 12:00 midnight) using Luxmeter, on a horizontal plane at participants’ eye level [19].

### 2.4. Bioanlytical Procedure

A total of 20 blood samples were collected from each participant at the following time points: 00.00 h (6 mL; pre-dose blood sample, within 60 min prior to dosing), 00.25, 00.50, 00.75, 01.00, 01.33, 01.67, 02.00, 02.33, 02.67, 03.00, 04.00, 05.00, 06.00, 07.00, 08.00, 10.00, 12.00, 14.00, and 24.00 h (5 mL, post-dose blood sample). All blood samples were collected in pre-labelled tubes filled with anticoagulant (dipotassium ethylenediaminetetraacetic acid [K_2_EDTA]) and centrifuged at 3800 rpm for 10 min at 4 °C (Centrifuge 5804 R) within 30 min of the collection of samples (at each collection time-point) to separate the plasma. The plasma samples were transferred to pre-labelled polypropylene tubes into two aliquots (approximately 1.2 mL in the first aliquot [Aliquot-1] and the remaining plasma in the second aliquot [Aliquot-2]) and stored in a freezer at a temperature of −30 ± 10 °C for interim storage at the clinical site and then transferred to the analytical site after the completion of the clinical phase for bioanalytical study.

A sensitive and selective quantification method was used to quantify the melatonin concentration in plasma by using ultra-high performance liquid chromatography–mass spectroscopy (UHPLC-MS/MS) in the bioanalytical facility of Spinos Life Science and Research Pvt. Ltd., Coimbatore, India. Plasma concentrations of melatonin over the concentration range of 201.30 to 200,629.90 pg/mL were estimated quantitatively using an optimized and validated LC-MS/MS method. Melatonin D4 was used as an internal standard. The reference standards melatonin (99.34% purity) and melatonin D4 (99.45% purity) were purchased from Vivan Life Sciences, Mumbai, India. Prior to use, the melatonin standard was stored at 2–8 °C, and melatonin D4 standard was stored at −20 °C. Melatonin and melatonin D4 were selectively extracted from the plasma sample by a solid phase extraction technique. Melatonin and melatonin D4 were separated using Strata-X 33 μm (Phenomenex, Torrance, CA, USA) Polymeric Reversed Phase cartridges. Chromatographic separation was achieved by reverse phase liquid chromatography on ZORBAX Eclipse XDB-C18 (4.6 mm × 100 mm, Agilent Technologies, Santa Clara, CA, USA), 3.5 μm column maintained at 45 °C. Internal standard was added to all the samples except the blank and the samples were then further analyzed for melatonin concentration using API 4000 LC-MS/MS system (AB Sciex, Framingham, MA, USA) detector, LC30AD (Shimadzu, Kyoto, Japan) pump, SIL30AC (Shimadzu, Kyoto, Japan) autosampler, CTO20AC (Shimadzu, Kyoto, Japan) column oven, and analyst software version 1.6.2 (AB Sciex, Framingham, MA, USA) data acquisition system. Details of quantitative analysis of melatonin are provided in the Supplementary Materials.

#### 2.4.1. Calibration Curve

Calibration curves of melatonin were constructed by spiking standard solutions in human blank plasma samples to obtain nine calibration standards at 201.60, 504.20, 4400.00, 8747.50, 17,495.00, 34,990.10, 69,980.20, 139,960.30, and 199,943.30 pg/mL. Calibration curves were analyzed individually using a least square-weighted linear regression (1/X^2^) and regression values (r^2^) were >0.98. Using an optimized method, the lower limit of quantification (LLOQ) observed for melatonin in human plasma was 201.30 pg/mL.

#### 2.4.2. Bioanalytical Method Validation

The International Council for Harmonisation Bioanalytical Method Validation and Study Sample Analysis M10 (ICH M10) and the Bioanalytical Method Validation Guidance for Industry by the United States Food and Drug Administration (USFDA) were followed for the development of a validation protocol of the bioanalytical method [20,21]. The optimized method was validated for specificity, selectivity, sensitivity, precision, accuracy, and recovery and all the parameters were within the acceptance criteria.

### 2.5. Pharmacokinetic Evaluations

Primary pharmacokinetic parameters including C_max_, area under the curve from time 0 to the last quantifiable time-point (AUC_0–t_) and the area under curve from time 0 to infinity (AUC_0–∞_), and secondary pharmacokinetic parameters including T_max_, elimination rate constant (K_el_), t_½_, and AUC__%Extrap_obs_ were determined for both products.

### 2.6. Safety Assessment

Safety assessment was measured by assessing the number of adverse events (AEs) reported for Melatonin-SR and Melatonin-IR. The participants were closely observed by study personnel for any AEs and serious adverse events (SAEs) during and after the completion of the study. The AEs were categorized based on their severity as mild, moderate, or severe.

### 2.7. Statistical Analysis

Descriptive statistics (arithmetic mean, geometric mean, standard deviation [SD], coefficient of variation [CV], minimum, median, and maximum) for all parameters were calculated. The sample size for this study was determined by referring to the guidelines of the Central Drugs Standard Control Organization (CDSCO) which recommend including a minimum of 16 subjects in the bioavailability studies. The 90% confidence interval (CI) and the ratio of geometric least square means for the mean concentrations of melatonin present in both products was calculated for log-transformed parameters (C_max_, AUC_0–t_, and AUC_0–∞_). The log-transformed pharmacokinetic parameters were subjected to an analysis of variance (ANOVA) test followed by Dunnett’s test (student’s *t*-test) to determine the statistical significance between two products at a significance level of 5%. A *p*-value < 0.05 was considered significant. Statistical analysis was performed using a non-compartmental model of SAS^®^ software (SAS Institute Inc., Cary, NC, USA) on Windows, version 9.4.

## 3. Results

### 3.1. Demographic Details

**Figure 1:**
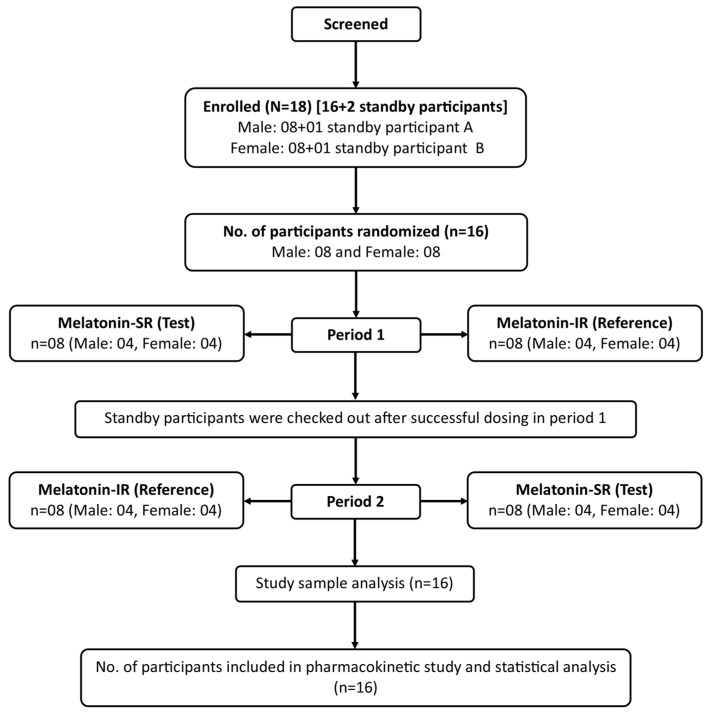
Disposition of the participants.

Sixteen healthy participants (eight men and eight women) who met all the eligibility criteria were enrolled in the study (Figure 1). All the participants successfully completed the study and were administered Melatonin-SR and Melatonin-IR in both study periods. The demographic details of the study participants are summarized in Table 1. The mean (SD) age and mean (SD) BMI of the participants was 33.25 (4.45) years and 25.48 (3.46) kg/m^2^, respectively (Table 1).

### 3.2. Pharmacokinetic Parameters

Following oral administration, the mean (SD) C_max_ of the test product (Melatonin-SR) was lower (11,446.87 [7804.98] pg/mL) as compared to the reference product (Melatonin-IR) (22,786.30 [13,772.70] pg/mL) (Table 2).

The analysis of secondary pharmacokinetic parameters is summarized in Table 3. The test product reported a higher mean T_max_ as compared to the reference product (1.26 [0.59] h vs. 0.87 [0.42] h). The mean (SD) t_1/2_ of the reference product (1.01 [0.68] h) was lower compared to the test product (5.10 [4.38] h) (Table 3).

**Figure 2:**
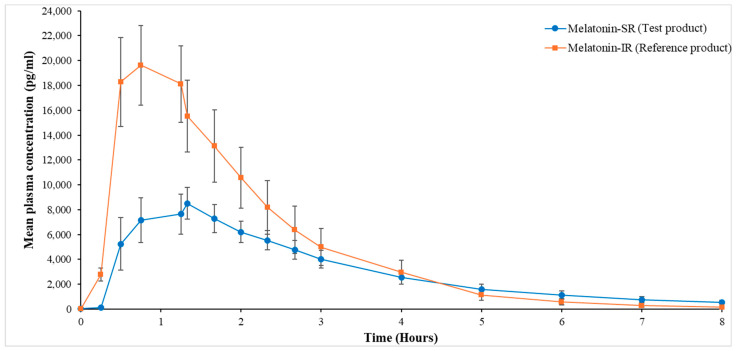
Mean plasma concentration vs. time curve for test and reference product from 0 to 8 h. All values are presented as mean (SEM). SEM, standard error of mean.

Clear differences in the release profiles of both the melatonin products were observed. In the initial phase (0–4 h), the plasma concentration of melatonin in the test product was lower as compared to that in the reference product. However, in the delayed phase (4–8 h), the plasma concentration of melatonin in the test product was higher as compared to that in the reference product (1.39- to 3.14-fold increase) for all time points until 8 h (Figure 2).

Particularly, in the test group, there was a gradual decrease in the melatonin concentration at timepoints 5, 6, 7, and 8 h from the 4 h timepoint, i.e., 1.59, 2.22, 3.30, and 4.74 times, respectively, as compared to the reference group, which showed a 2.57 and 5.07 times reduction in melatonin concentration at 5 h and 6 h, with a sharp decline of 10.32 and 18.69 times at 7 h and 8 h, respectively. These observed differences between the test and reference products in the melatonin concentration in the delayed phase are noteworthy though not significant (*p* > 0.05).

### 3.3. Safety Assessment

One AE of a single episode of vomiting was reported in one female participant during the study in period 1, at around 5 h after administration of the reference product (Melatonin-IR). This AE was mild and was considered as possibly related to the product. The participant had normal vitals, and general and systematic examinations. The participant was followed up for vital signs and physical and general clinical examinations until the next day. All the parameters were normal throughout the observation period, and the AE was considered resolved by the principal investigator. No other AEs or SAEs were reported by any other participants.

## 4. Discussion

The present study evaluated the pharmacokinetic profile of Melatonin-SR versus Melatonin-IR in healthy adults under the fasting condition. The key observations of this pharmacokinetic study were lower C_max_, higher T_max_, and a five-fold prolonged half-life of melatonin in the test product compared to that in the reference product. These results indicate the sustained-release pharmacokinetic profile of the test product Melatonin-SR.

Various publications have reported the pharmacokinetic profile of melatonin [7,14,16,19,22,23,24,25]. A phase 3, randomized, cross-over study conducted on 12 healthy subjects under fasting conditions showed a mean C_max_ of 5766 pg/mL, mean T_max_ of 60.3 min, and mean t_1/2_ of 65.0 min following a 4 mg oral administration of melatonin [23]. In the present study, the test product Melatonin-SR showed higher C_max_, prolonged T_max_, and longer t_1/2_ as compared to these results. A pharmacokinetic profile of melatonin reported by Gooneratne et al. (2012) was similar to the present study. It was a randomized, double-blind, placebo-controlled study which included 27 patients with insomnia who received 4 mg of an oral sustained-release product of melatonin and reported a T_max_ of 1.5 h, mean elimination half-life of 2.1 h, and mean C_max_ of 3999 (700) pg/mL [22]. Further, in the present study, the sustained-release profile of the test product was evident by the higher plasma concentration in the delayed phase from 4 to 8 h as compared to the reference product. This may have implications in minimizing the dosing frequency of melatonin in participants with sleeping disorders [23,25].

The role of melatonin in the induction and maintenance of sleep is well documented [26,27]. It is believed that patients with insomnia and other sleep disorders may lack N-acetyltransferase, an enzyme involved in the conversion of melatonin into its hypnotic derivative, which may help in the induction and maintenance of sleep [27]. Melatonin plasma concentrations in adults typically range from 20 to 120 pg/mL in the nighttime, which might reach as high as 160 pg/mL. Notably, around 80% of melatonin production occurs between 12 a.m. and 4 a.m. The high concentration of melatonin during these midnight hours may help to maintain sound sleep in humans [13,28,29]. In our study, the mean plasma concentration of melatonin following the test product administration was maintained above the normal range in healthy adults. This indicated that the melatonin released through the test product remained in the plasma for a longer duration, which can help induce and maintain sound sleep for long hours in healthy adults. This can also be beneficial for adults with an abnormal sleep cycle, who are on shift work, and for those who are badly affected by jet lag.

Delayed sleep–wake phase disorder is considered as the most common of the circadian rhythm sleep–wake disorders, which are an increasingly recognized and diagnosed group of sleep disorders. These disorders are primarily caused by external factors like jet lag or shift work. Melatonin formulations are widely used in these cases but not without limitations such as quick clearance, low plasma concentration, shorter half-life, and shorter T_max_ [30]. Consequently, these patients often face high pill burden to manage their symptoms. In our study, the test product showed a prolonged T_max_, longer half-life, and maintained a high level of plasma concentration for 8 h compared to the reference product, which in turn can help in minimizing dosing frequency in these sleep disorders. In the Melatonin-IR group, we observed a decline in the plasma levels of melatonin after 0.75 h; however, in the melatonin-SR group, this decline was observed at a later time point, i.e., after 1.33 h, which gradually continued up to 8 h. Although, this decrease in melatonin levels was observed in both groups, the rate at which melatonin levels declined in the Melatonin-SR group was slower than that of the Melatonin-IR group. In particular, in the delayed phase, i.e., after 4 h till 8 h, the plasma levels of melatonin in the Melatonin-SR group were 1.39- to 3.41-fold higher than the Melatonin-IR group. These observations indicate that the SR formulation provides a steady and slow release of melatonin throughout the 8 h sleep period. Additionally, this gradual decline in plasma melatonin levels in the body would further help maintain consistent sleep throughout the night without compromising morning alertness [31]. Given the limitations of exogenous melatonin in IR formulations, such as the short elimination half-life, which can disrupt sleep, the SR formulation offers a superior alternative. With its extended half-life and continuous, gradual release of melatonin over a longer duration, the SR formulation would be effective in facilitating a peaceful and longer sleep while maintaining a balanced sleep–wake cycle [13,14,30].

In our study, one participant reported an AE following the administration of the reference product; it was of mild severity, short-lived, and resolved on its own. Therefore, both the products were considered as well tolerated and safe for oral administration. The safety results of an open-label, two-way, crossover, randomized study, comparing continuous release and absorption melatonin (melatonin-CRA) (5 mg) vs. melatonin-IR (5 mg), reported treatment emergent AEs (including emesis, stomach cramps, nausea, and irritability) in patients who received melatonin-IR and no AEs in the melatonin-CRA group. These results are in line with the present study [18]. Furthermore, Foley and Steel, in their systematic review (included 50 clinical studies), documented AEs associated with melatonin oral administration. The most common AEs were related to fatigue, mood, or psychomotor and neurocognitive performance which were generally short-lived and associated with day-time dosing [32].

The limitations of this study include a small sample size of 16 healthy participants and a single dose study. However, large-scale studies with multiple doses administered over multiple days or weeks on a larger participant pool from varied demographic backgrounds can help obtain a more real-world pharmacokinetic profile of the newly developed Melatonin-SR capsules. Moreover, the notable difference in the melatonin levels during the delayed phase (4 h till 8 h, post-administration exogenous melatonin formulations) between SR and IR formulations can serve as potential pointer to further evaluate the impact of these differences on sleep quality in healthy individuals through well-designed clinical trials. These studies will help validate the pharmacokinetic differences observed and their implications for sleep management.

## 5. Conclusions

Melatonin-SR capsules, on a single oral dose administration, elevated plasma melatonin levels and sustained them over an extended period (up to 8 h) as compared to immediate release products. Melatonin-SR was safe and well-tolerated. Thus, Melatonin-SR may be considered as a promising nutraceutical supplement for promoting and maintaining healthy sleep patterns, especially for adults grappling with erratic sleep cycles.

## Figures and Tables

**Table 1:** Demographic characteristics of the participants.

Characteristic	Mean (SD)	Coefficient of Variance (%)	Range
Age (years)	33.25 (4.45)	13.38	26.00–40.00
Height (cm)	162.60 (6.22)	3.83	153.00–173.70
Weight (kg)	67.18 (8.48)	12.62	53.80–81.50
BMI (kg/m^2^)	25.48 (3.46)	13.58	18.84–29.59

N = 16; male: 08; female: 08; BMI, body mass index; SD, standard deviation.

**Table 2:** Comparison of primary pharmacokinetic parameters between test and reference products.

Parameters	Mean (SD)		Geometric Least Square Mean				
	Test (T)	Reference (R)	Test (T)	Reference (R)	Ratio of T/R	90% CI	*p*-Value
C_max_ (pg/mL)	11,446.87 (7804.98)	22,786.30 (13,772.70)	9681.86	19,587.29	49.43	38.32–63.77	*p* < 0.05
AUC_0–t_ (h×pg/mL)	30,004.59 (21,291.97)	43,078.8322 (35,854.20)	24,065.72	33,483.50	71.87	63.16–81.79	*p* < 0.05
AUC_0–∞_ (h×pg/mL)	35,812.98 (28,295.06) ^a^	43,539.37 (37,146.72) ^b^	24,565.12	34,464.67	71.28	61.41–82.73	*p* < 0.05

^a^ Five participants were excluded from the calculation of AUC_0–∞_ as the adjusted R-square value was less than 0.8. ^b^ One participant was excluded from the calculation of AUC_0–∞_ as the adjusted R-square value was less than 0.8. AUC_0–t_, area under curve from time 0 to the last quantifiable time-point; AUC_0–∞_, area under curve from time 0 to infinity; CI, confidence interval; C_max_, maximum drug concentration in plasma; reference, Melatonin-IR 5 mg immediate-release melatonin capsules; SD, standard deviation; test, Melatonin-SR 5 mg sustained-release melatonin capsules.

**Table 3:** Secondary pharmacokinetic parameters of test and reference products.

Parameters	Test (T)	Reference (R)
T_max_ (h)	1.26 (0.59)	0.87 (0.42)
K_el_ (1/h)	0.36 (0.33) ^a^	0.84 (0.29) ^b^
t_½_ (h)	5.10 (4.38) ^a^	1.01 (0.68) ^b^
AUC__%Extrap_obs_ (%)	7.66 (4.54) ^a^	1.84 (1.26) ^b^

All values are presented as mean (SD). ^a^ Five participants were excluded from the calculation of K_el_, t_1/2_, and AUC__%Extrap_obs_ as the adjusted R-square value was less than 0.8. ^b^ One participant was excluded from the calculation of K_el_, t_1/2_, and AUC__%Extrap_obs_ as the adjusted R-square value was less than 0.8. K_el_, elimination rate constant; reference, Melatonin-IR 5 mg immediate-release melatonin capsules; SD, standard deviation; test, Melatonin-SR 5 mg sustained-release melatonin capsules; T_max_, time to reach maximum concentration in plasma; t_1/2_, elimination half-life.

## Data Availability

The datasets used for this study will be available from the corresponding author upon reasonable request.

## Supplementary Materials

The following supporting information can be downloaded at: https://www.mdpi.com/article/10.3390/pharmaceutics16101248/s1. Quantitative analysis of melatonin by ultra-high performance liquid chromatography-mass spectroscopy.

## References

[1] Irwin M.R. (2015). Why sleep is important for health: A psychoneuroimmunology perspective. Annu. Rev. Psychol..

[2] Abbott S.M., Videnovic A. (2016). Chronic sleep disturbance and neural injury: Links to neurodegenerative disease. Nat. Sci. Sleep.

[3] Ali T., Choe J., Awab A., Wagener T.L., Orr W.C. (2013). Sleep, immunity and inflammation in gastrointestinal disorders. World J. Gastroenterol..

[4] Kim T.W., Jeong J.H., Hong S.C. (2015). The impact of sleep and circadian disturbance on hormones and metabolism. Int. J. Endocrinol..

[5] Medic G., Wille M., Hemels M.E. (2017). Short- and long-term health consequences of sleep disruption. Nat. Sci. Sleep.

[6] Tasali E., Leproult R., Ehrmann D.A., Van Cauter E. (2008). Slow-wave sleep and the risk of type 2 diabetes in humans. Proc. Natl. Acad. Sci. USA.

[7] Wade A.G., Ford I., Crawford G., Mcconnachie A., Nir T., Laudon M., Zisapel N. (2010). Nightly treatment of primary insomnia with prolonged release melatonin for 6 months: A randomized placebo-controlled trial on age and endogenous melatonin as predictors of efficacy and safety. BMC Med..

[8] Gordon N.P., Yao J.H., Brickner L.A., Lo J.C. (2022). Prevalence of sleep-related problems and risks in a community-dwelling older adult population: A cross-sectional survey-based study. BMC Public Health.

[9] Adjaye-Gbewonyo D., Ng A.E., Black L.I. Sleep Difficulties in Adults: United States, 2020 Key Findings Data from the National Health Interview Survey. https://www.cdc.gov/nchs/products/index.htm.

[10] National Health Service Report. Part of Mental Health of Children and Young People Surveys, Mental Health of Children and Young People in England 2022—Wave 3 Follow Up to the 2017 Survey, England, Published on 29 November 2022. https://digital.nhs.uk/data-and-information/publications/statistical/mental-health-of-children-and-young-people-in-england/2022-follow-up-to-the-2017-survey/part-2-sleep-loneliness-and-health-behaviours.

[11] Bhaskar S., Hemavathy D., Prasad S. (2016). Prevalence of chronic insomnia in adult patients and its correlation with medical comorbidities. J. Fam. Med. Prim. Care.

[12] Cardinali D.P., Pévet P. (1998). Basic aspects of melatonin action. Sleep Med. Rev..

[13] Tordjman S., Chokron S., Delorme R., Charrier A., Bellissant E., Jaafari N., Fougerou C. (2017). Melatonin: Pharmacology, functions and therapeutic benefits. Curr. Neuropharmacol..

[14] Harpsøe N.G., Andersen L.P.H., Gögenur I., Rosenberg J. (2017). Clinical pharmacokinetics of melatonin: A systematic review. Eur. J. Clin. Pharmacol..

[15] Härtter S., Nordmark A., Rose D.M., Bertilsson L., Tybring G., Laine K. (2003). Effects of caffeine intake on the pharmacokinetics of melatonin, a probe drug for CYP1A2 activity. Br. J. Clin. Pharmacol..

[16] Hilli J., Korhonen T., Turpeinen M., Hokkanen J., Mattila S., Laine K. (2008). The effect of oral contraceptives on the pharmacokinetics of melatonin in healthy subjects with CYP1A2 g.-163C> A Polymorphism. J. Clin. Pharmacol..

[17] Ursing C., von Bahr C., Brismar K., Röjdmark S. (2005). Influence of cigarette smoking on melatonin levels in man. Eur. J. Clin. Pharmacol..

[18] Potes Y., Cachán-Vega C., Antuña E., García-González C., Menéndez-Coto N., Boga J.A., Gutiérrez-Rodríguez J., Bermúdez M., Sierra V., Vega-Naredo I. (2023). Benefits of the Neurogenic Potential of Melatonin for Treating Neurological and Neuropsychiatric Disorders. Int. J. Mol. Sci..

[19] Seiden D.J., Shah S.M. (2019). A randomized, crossover, pharmacokinetics evaluation of a novel continuous release and absorption melatonin formulation. Prim. Care Companion CNS Disord..

[20] The Bioanalytical Method Validation Guidance for Industry USFDA 2018. https://www.fda.gov/files/drugs/published/Bioanalytical-Method-Validation-Guidance-for-Industry.pdf.

[21] International Council for Harmonisation of Technical Requirements for Pharmaceuticals for Human Use: Bioanalytical Method Validation and Study Sample Analysis M10 (ICH M10) (Adopted on 24 May 2022). https://database.ich.org/sites/default/files/M10_Guideline_Step4_2022_0524.pdf.

[22] Gooneratne N.S., Edwards A.Y.Z., Zhou C., Cuellar N., Grandner M.A., Barrett J.S. (2012). Melatonin pharmacokinetics following two different oral surge-sustained release doses in older adults. J. Pineal Res..

[23] Demuro R.L., Nafziger A.N., Blask D.E., Menhinick A.M., Bertino J.S. (2000). The absolute bioavailability of oral melatonin. J. Clin. Pharmacol..

[24] Gandolfi J.V., Bernardo A.P.A.D., Chanes D.A.V., Martin D.F., Joles V.B., Amendola C.P., Sanches L.C., Ciorlia G.L., Lobo S.M. (2020). The effects of melatonin supplementation on sleep quality and assessment of the serum melatonin in ICU patients: A randomized controlled trial. Crit. Care Med..

[25] Román Martinez M., García Aguilar E., Martin Vílchez S., González García J., Luquero-Bueno S., Camargo-Mamani P., Mejia-Abril G., García-Castro L., de Miguel-Cáceres A., Saz-Leal P. (2022). Bioavailability of Oniria^®^, a melatonin prolonged-release formulation, versus immediate-release melatonin in healthy volunteers. Drugs R D.

[26] Poza J.J., Pujol M., Ortega-Albás J.J., Romero O., En Representación del Grupo de Estudio de Insomnio de la Sociedad Española de Sueño (SES) (2022). Melatonin in sleep disorders. Melatonina en los trastornos de sueño. Neurologia.

[27] Fourtillan J.B. (2002). Role of melatonin in the induction and maintenance of sleep. Dialogues Clin. Neurosci..

[28] Brzezinski A. (1997). Melatonin in humans. N. Engl. J. Med..

[29] Karasek M. (2004). Melatonin, human aging, and age-related diseases. Exp. Gerontol..

[30] Nesbitt A.D. (2018). Delayed sleep-wake phase disorder. J. Thorac. Dis..

[31] Watson N.F., Badr M.S., Belenky G., Bliwise D.L., Buxton O.M., Buysse D., Dinges D.F., Gangwisch J., Grandner M.A., Kushida C. (2015). Recommended amount of sleep for a healthy adult: A joint consensus statement of the American Academy of Sleep Medicine and Sleep Research Society. Sleep.

[32] Foley H.M., Steel A.E. (2019). Adverse events associated with oral administration of melatonin: A critical systematic review of clinical evidence. Complement. Ther. Med..

