# Supplementary material for: Comparative Pharmacokinetics of Sustained-Release versus Immediate-Release Melatonin Capsules in Fasting Healthy Adults: A Randomized, Open-Label, Cross-Over Study
Source: Pharmaceutics. 2024 Sep 25;16(10):1248. doi: 10.3390/pharmaceutics16101248 (PMC11510348; doi:10.3390/pharmaceutics16101248)
Supplement: Supplementary file 1 [file pharmaceutics-16-01248-s001.zip › pharmaceutics-3132307-supplementary.pdf]

**Supplementary Material**

**Quantitative analysis of melatonin by ultra-high performance liquid chromatography-mass spectroscopy (UHPLC-MS/MS):**

*Sample Solution Preparation*

Plasma was obtained by centrifuging $K_2EDTA$ blood samples at 3,800 rpm for 10 min at 4°C. Thereafter, a total of 300 µL of plasma was added to 50 µL of working solution (100 ng/mL) of the melatonin D4 and 300 µL of buffer-02 (20 mM disodium hydrogen orthophosphate [$Na_2HPO_4$] in water) was added to each sample and vortexed. Melatonin and melatonin D4 were separated using Strata-X 33 µm (Phenomenex, Torrance, USA) Polymeric Reversed Phase cartridges. Briefly, cartridges were activated with 1 mL of acetone-M and subsequently washed with 1 mL of HPLC grade water. Thereafter, samples were loaded on the cartridges and centrifuged at 1500 rpm for 2 min. Further, cartridges were washed with 1 ml of HPLC water followed by 1 mL of washing solution of 5% acetone-M in water (5:95). Cartridges were dried for 5 min at 3500 rpm and 300 µL of elution solution (acetonitrile: water [90:10] v/v with 0.1% formic acid in water) was added to the dried cartridge and centrifuged at 1500 rpm for 2 min to elute out the samples. Eluted samples were transferred into respectively labelled 1.5 mL microcentrifuge tubes and centrifuged at 14000 rpm for 5 min. These were further transferred into labelled injector vials and injected into the UHPLC-MS/MS system for analysis.

*Stock Solution preparation*

Stock solutions were prepared at 1 mg/mL by dissolving melatonin and melatonin D4 (in acetone-M). The working solution for internal standard was prepared at 100 ng/mL by diluting the corresponding stock solution in acetone-M.

*Chromatographic Condition*

The liquid chromatography (LC) system consisted of an UHPLC (Shimadzu). Chromatographic separation was achieved by reverse phase liquid chromatography on

ZORBAX Eclipse XDB-C18 (4.6 mm x 100 mm, Agilent technologies, USA), 3.5 µm column maintained at 45ºC. For regulation of temperature of the column, CTO20AC column oven (from Shimadzu, Japan) was used. The mobile phase was delivered (using LC30AD [Shimadzu, Japan] pump) at a flow rate of 0.6 mL/min through low pressure gradient and consisted of 80% acetonitrile (organic mobile phase A) and 20% buffer-01 (0.1% formic acid in water v/v) (aqueous mobile phase B). The analytical run time for each injection was 3.5 min. A total of 10 µL sample was injected in UHPLC-MS/MS system using SIL30AC (Shimadzu, Japan) autosampler for quantification of melatonin.

*Mass Spectrometric Condition*

Detection of melatonin and melatonin D4, based on the peaks' mass to charge (m/z) ratio, was carried out using a Mass Spectrometry system (AB Sciex) equipped with a source operating API 4000 in the positive ion (ESI+) mode. Mass spectrometric conditions were as follows: Ion Spray Voltage 5500, sheath Gas temperature 400°C, Curtain gas flow rate of 15 psi, Charged aerosol detector of 15 psi, ion source gas (GS1 of 45 psi, and GS2 of 55 psi). Samples were detected in multiple reaction monitor mode. For data acquisition and analysis, the Analyst software version 1.6.2 (supplied by AB Sciex) was used.

*Details of the precision and accuracy data for the assay:*

Assay precision and accuracy values were determined across one precision and accuracy batch by analyzing six replicates of each QC samples (Limit of Quantification Quality Control [LOQQC], Lower Quality Control [LQC], Intermediate Quality Control [INTQC], Middle Quality Control [MQC], and Higher Quality Control [HQC]) in each batch. The precision and accuracy determined using these QC samples were found to be within acceptable range (LOQC samples: ≤20% and 80-120%; Other QC samples of nominal concentration: ≤15% and 85-115%, respectively). The precision of the assay was measured by the percentage co-efficient of variation over the concentration range of QC samples of

Melatonin during the course of partial method validation. The total precision of Melatonin at LOQQC was 7.86% and for other QC samples (LQC, INTQC, MQC and HQC), it ranged from 1.85% to 7.78%. The accuracy of the assay is defined as the absolute value of the ratio of the calculated mean values of the QC samples to their respective nominal values expressed as percentage. The batch accuracy of Melatonin for LOQQC was 95.44%. and for other QC samples (LQC, INTQC, MQC and HQC), it ranged from 92.16% to 102.60%.
